# A stage IIIA lung adenocarcinoma case achieving pathological response with only one cycle of preoperative nivolumab combination chemotherapy

**DOI:** 10.1186/s44215-025-00187-5

**Published:** 2025-02-03

**Authors:** Shuhei Baba, Fumihiko Kinoshita, Yoshihiro Yamamoto, Yoshiyuki Nakanishi, Takaki Akamine, Mikihiro Kohno, Keigo Ozono, Tomoyoshi Takenaka, Tomoharu Yoshizumi

**Affiliations:** 1 Department of Surgery and Science, Graduate School of Medical Sciences, Kyushu University, Fukuoka, Japan; 2 Department of Thoracic Surgery, Kyushu University Hospital, 3-1-1 Maidashi, Higashi-Ku, Fukuoka, 812-8582 Japan; 3 Department of Respiratory Medicine, Graduate School of Medical Sciences, Kyushu University, Fukuoka, Japan; 4 Department of Anatomic Pathology, Graduate School of Medical Sciences, Kyushu University, Fukuoka, Japan

**Keywords:** Neoadjuvant therapy, Surgery, Lung cancer

## Abstract

**Background:**

Preoperative nivolumab combination chemotherapy has shown its efficacy in resectable stage II–III non-small cell lung cancer and become one of the standard treatments. While preoperative nivolumab combination chemotherapy is generally a regimen of three cycles, the efficacy of nivolumab combination chemotherapy when treatment is prematurely discontinued remains unclear.

**Case presentation:**

An 81-year-old man was diagnosed as lung adenocarcinoma (cT3N1M0, cStage IIIA). A computed tomography (CT) showed a 58 mm mass in left upper lobe with an intrapulmonary metastasis, and a positron-emission tomography/CT suggested metastatic lymph nodes at the left pulmonary hilum. Preoperative nivolumab + carboplatin + paclitaxel were administered; however, after the first cycle, the treatment was discontinued due to grade 3 anorexia, grade 1 body weight loss, and grade 4 neutropenia. It was affair that continuation of preoperative therapy made him unsuitable for surgery, and CT scan showed a reduction in the tumor size to 20 mm. Then, we decided to discontinue the preoperative therapy and perform surgery. Video-assisted thoracoscopic left upper lobectomy and lymph node dissection were performed, and the postoperative course was uneventful. The pathological examination revealed 15% of residual tumor cell in primary lesion and no metastatic lymph nodes was diagnosed. The patient did not undergo adjuvant chemotherapy, and no recurrence was observed 1.5 years after surgery

**Conclusions:**

In this case, preoperative nivolumab combined chemotherapy was discontinued only one cycle due to adverse events; however, a significant treatment effect was achieved. Therefore, even it is unable to continue preoperative nivolumab combined therapy, it is important not to miss the chance of surgery, as good treatment effect may have been achieved.

**Supplementary Information:**

The online version contains supplementary material available at 10.1186/s44215-025-00187-5.

## Background

Preoperative immune checkpoint inhibitor (ICI) combination chemotherapy has demonstrated efficacy in resectable stage II–III non-small cell lung cancer (NSCLC) and has become one of the standard treatments [1–9]. The CheckMate-816 trial demonstrated improvements in the primary endpoints of event-free survival (EFS) and pathological complete response (pCR) with preoperative nivolumab combination chemotherapy (median EFS: 31.6 vs. 20.8 months, *p* = 0.005; pCR: 24% vs. 2.2%, *p* < 0.001) [1]. Similar results supporting the incorporation of ICI in perioperative chemotherapy for stage II–III NSCLC were observed in several phase III clinical trials, such as the CheckMate-77 T trial [2], KEYNOTE-671 trial [3], and the AEGEAN trial [4].

In clinical trials, preoperative ICI combination chemotherapy is generally designed as a regimen consisting of three or four cycles [1–4]. However, in clinical practice, the completion of preoperative treatment is often compromised due to adverse events. The therapeutic efficacy of preoperative ICI combination chemotherapy when treatment cannot be continued due to adverse events remains unclear.

Herein, we present a case in which, although preoperative nivolumab combination chemotherapy was discontinued after only one cycle owing to serious side effects, a marked therapeutic effect was observed.

## Case presentation

**Fig. 1:**
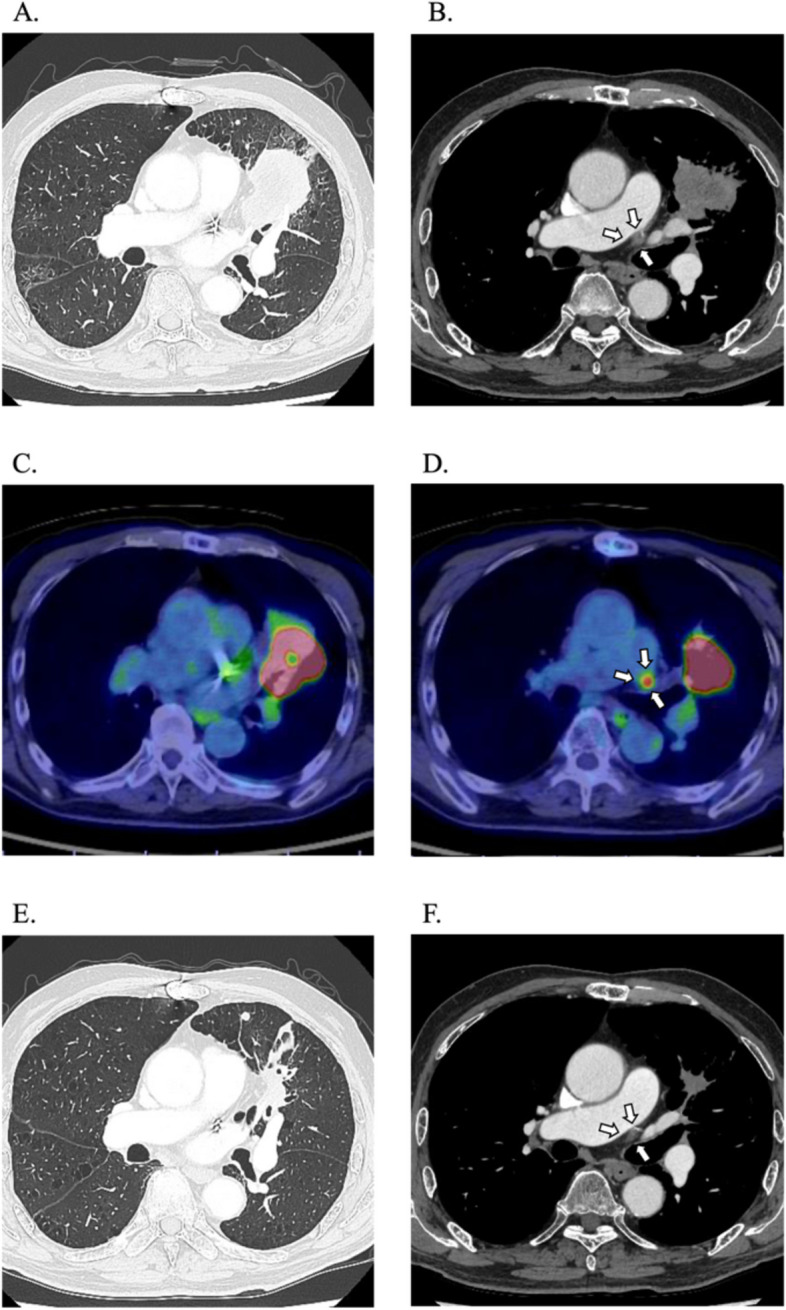
Computed tomography (CT) scan of primary lesion (**A**) and left hilar lymph node (**B**) before neoadjuvant nivolumab combination chemotherapy. Positron-emission tomography/CT of primary lesion (**C**) and left hilar lymph node (**D**) before neoadjuvant nivolumab combination chemotherapy. CT scan of primary lesion (**E**) and left hilar lymph node (**F**) after one cycle of neoadjuvant combination chemotherapy

An 81-year-old man developed symptoms including an upper respiratory tract infection and sought medical attention at a local hospital to determine the cause. His performance status (PS) was 1. He underwent aortic valve replacement (bioprosthesis), mitral valve replacement (bioprosthesis), tricuspid annuloplasty, and left atrial appendage closure via median sternotomy for moderate aortic regurgitation, severe mitral regurgitation, and moderate tricuspid regurgitation 3 years ago. Computed tomography (CT) revealed a 58 mm mass in the left upper lobe (LUL, Fig. 1A). Additionally, other small nodules suggestive of intrapulmonary metastases were also identified in the LUL (cT3[PM1], Fig. 1A). The left hilar lymph node was mildly enlarged (Fig. 1B). Positron emission tomography/CT (PET/CT) revealed abnormal accumulation at the tumor in the LUL with a standard uptake value-max (SUV-max) of 17.01 (Fig. 1C). Moreover, PET/CT demonstrated abnormal accumulation in the lymph nodes at the left pulmonary hilum with SUV-max of 5.65, raising suspicion of lymph node metastasis (cN1, Fig. 1D). A bronchoscopic biopsy of the primary lesion confirmed lung adenocarcinoma, and the patient was diagnosed with primary lung adenocarcinoma (cT3[PM1] N1M0, cStage IIIA). Immunohistochemical staining exhibited that the expression of programmed cell death-ligand 1 protein in the primary tumor ranged from 1 to 24%. No driver mutations, including *epithelial growth factor receptor* mutations and *anaplastic lymphoma kinase* rearrangements, were detected by AmoyDx lung cancer multi-gene PCR panel test.

Regarding the patient’s general condition, despite his history of cardiac surgery, echocardiography showed left ventricular ejection fraction of 75%, no wall motion abnormalities, and no valve dysfunction. Therefore, his cardiac function was relatively well preserved. Furthermore, regarding respiratory function, spirometry showed a forced vital capacity (FVC) of 3.26 L (95.6% of predicted FVC) and a forced expiratory volume in one second (FEV1.0) of 2.41 L (73.9% of predicted FEV1.0). Based on these findings, we concluded that his surgical tolerance was adequate.

We decided to proceed with surgery after preoperative nivolumab combination chemotherapy. The regimen included nivolumab (360 mg/body), carboplatin (area under the curve = 6, 300.5 mg/body), and paclitaxel (200 mg/body and 329 mg/body). However, after the first cycle of nivolumab + carboplatin + paclitaxel, grade 3 anorexia, grade 1 body weight loss, and grade 4 neutropenia (neutrophil count, 498/mm^2^) were observed.

During the initial cycle of the treatment, a decline in performance status (PS) was noted. Furthermore, the patient experienced a weight loss of 4.6 kg (7.9%) compared to the weight at baseline. Despite the PS remaining at 1, concerns arose that continued administration of the first cycle of treatment led to a further decrease in PS, rendering the patient unsuitable for surgery. In addition, CT performed 17 days after the first administration demonstrated a significant reduction in the primary lesions and lymphatic metastasis. The tumor size decreased from 58 to 20 mm, leading us to classify the treatment response as a partial response (15%) (Fig. 1E). The left hilar lymph node was slightly reduced in size (Fig. 1F). Based on these considerations, we decided to discontinue neoadjuvant therapy and perform surgery. The preoperative diagnoses were ycT3[PM1] N1M0 and ycStage IIIA.

A video-assisted thoracoscopic left upper lobectomy and lymph node dissection were performed. An 8 cm lateral incision was made on the 4th intercostal space, and an assist port was placed at the anterior axillary line of 7th intercostal space. No pleural dissemination was noted within the thoracic cavity, although a small volume of serous pleural effusion was observed. Mild adhesions were observed between the LUL and the chest wall; however, no adhesions, fibrosis, or lymph node involvement were noted around the pulmonary artery, pulmonary vein, or bronchus at the pulmonary hilum that would interfere with surgery (Supplementary Fig. 1). Intraoperatively, a bleeding was encountered from a pulmonary artery (A3), which appeared unrelated to the preoperative treatment. The operation time was 210 min, with a total blood loss of 877 ml. The postoperative course was uneventful. The chest drain was removed on postoperative day 3, and the patient was discharged on postoperative day 12.

**Fig. 2:**
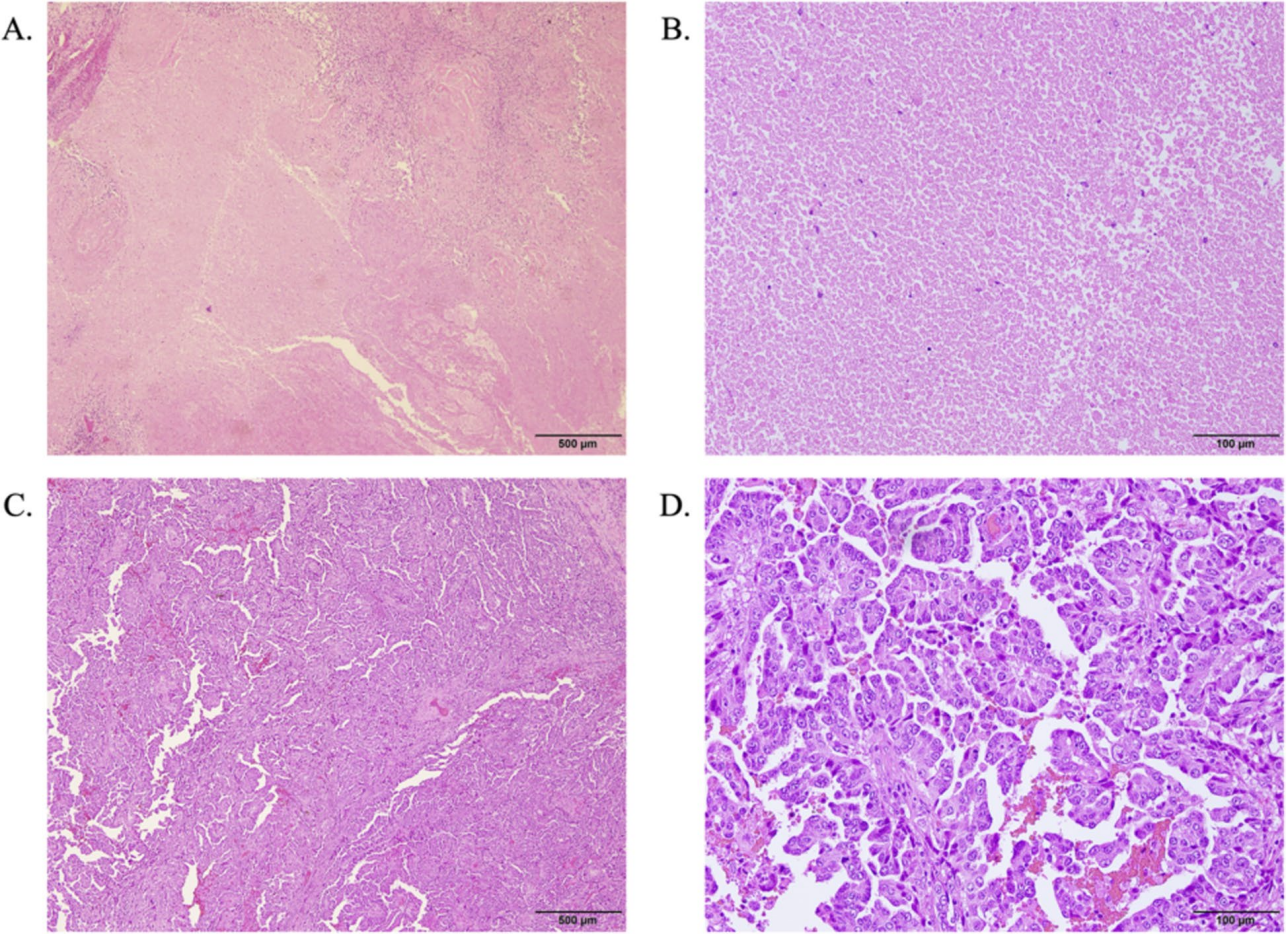
Pathological images of surgically resected specimens with a primary tumor of the left upper lobe. Images of necrotic tumor bed in 40 × (**A**) and 200 × (**B**) magnification. Images of residual tumor cells in 40 × (**C**) and 200 × (**D**) magnification

The pathological diagnosis was papillary-predominant adenocarcinoma (component: papillary 80%; lepidic 20%) (Fig. 2A and B). The total size of the primary tumor was 20 × 11 × 18 mm, while the invasive size was 9 × 5 × 7 mm. No pleural, lymph, or vascular invasions were observed. No intrapulmonary metastases were observed, with no evidence of lymph node metastasis (ypN0). The final diagnosis was ypT1aN0cM0 yp Stage IA1. The residual viable tumor cells accounted for 15%, and the effectiveness of neoadjuvant therapy was evaluated as Ef.2 (Fig. 2C and D). Pleural fluid cytology was negative for malignancy. In addition, the nodule, which was suspected as intrapulmonary metastasis in preoperative CT scan, was diagnosed as granulation tissue. The patient did not undergo adjuvant chemotherapy, and no recurrence was observed 1.5 years after surgery.

## Discussion and conclusions

The CheckMate-816 trial, a multicenter, international, non-blinded, randomized phase III trial, elucidated the efficacy of nivolumab combination chemotherapy as neoadjuvant therapy in patients with resectable stage IB–IIIA NSCLC [1]. Regarding pathological treatment response, patients who underwent three cycles of preoperative nivolumab combination chemotherapy followed by complete resection demonstrated a median proportion of residual tumor cells of 10% (interquartile range 0–10) [1]. In our case, only one cycle of neoadjuvant nivolumab combination chemotherapy achieved a residual tumor cell count of 15%, demonstrating a significant therapeutic effect.

In several clinical trials, including the CheckMate-816 trial, which demonstrated the efficacy of neoadjuvant ICI combination chemotherapy, a pCR or major pathological response (MPR) was associated with a favorable postoperative prognosis [1–3, 10]. Therefore, the pathological response to preoperative ICI combination chemotherapy is considered a predictor of postoperative prognosis [10]. In the present case, the residual viable tumor was 15%. Although pCR or MPR was not attained, a favorable postoperative prognosis might be expected.

In the CheckMate-816 trial, 5.7% (10/176) of patients in the preoperative nivolumab combination chemotherapy group discontinued treatment due to toxicity [1]. As cases in which preoperative nivolumab combined with chemotherapy was discontinued were excluded from the efficacy analysis, the treatment efficacy in cases unable to complete three courses of preoperative nivolumab combination chemotherapy remains uncertain [1].

Regarding the safety of preoperative treatment, the CheckMate-816 trial reported a 33.5% incidence of grade 3 or 4 treatment-related adverse events in the nivolumab combination chemotherapy group compared to 36.9% in the chemotherapy alone group [1]. Additionally, the incidences of adverse events leading to preoperative treatment discontinuation were 10.2% in the preoperative nivolumab combination chemotherapy group and 9.7% in the preoperative chemotherapy alone group [1]. The addition of nivolumab to preoperative chemotherapy did not lead to an increase in discontinuation. However, a certain probability exists that preoperative nivolumab combination chemotherapy would be discontinued in some cases.

In the CheckMate-816 trial, preoperative nivolumab combination chemotherapy was specified for three cycles, whereas in other trials, preoperative ICI combination chemotherapy was often prescribed for four cycles [1–4]. In contrast, several clinical trials have reported favorable outcomes with only two cycles of preoperative ICI combination therapy [11, 12]. The optimal number of cycles of preoperative ICI combination therapy requires further investigation. Consider that some cases may require only one cycle of treatment, as demonstrated in this report. Additionally, the opportunity for surgery may be lost due to disease progression or deterioration of the general condition during multiple cycles of preoperative treatment.

In advanced NSCLC, several reports have analyzed the effectiveness of ICI therapy with a limited number of cycles. An analysis of 17 patients with advanced NSCLC who discontinued nivolumab therapy due to reasons other than disease progression showed that 6 patients (35.3%) achieved a progression-free survival (PFS) of 6 months or longer without additional treatment [13]. Similarly, another study of 19 patients with NSCLC who discontinued ICI therapy due to adverse events demonstrated that the median PFS after discontinuation was 5.6 months [14]. In particular, patients who achieved partial response had a good median PFS of 10.2 months [14]. These findings suggest that ICI therapy including nivolumab may be effective even with a limited number of cycles in certain NSCLC patients.

The CheckMate-816 trial demonstrated that patients with nivolumab plus chemotherapy were more likely to undergo minimally invasive approach, such as video-assisted thoracoscopic surgery (VATS), compared to those with chemotherapy alone (*N* = 44/179 [29.5%] versus *N* = 29/179 [21.5%]) [1]. In the present case, despite the limited number of cycles of nivolumab plus chemotherapy, curative surgery could be performed via VATS. Therefore, our case was consistent with the findings of the CheckMate-816 trial, supporting the feasibility of minimally invasive surgical approach in cases with preoperative treatment.

In this case, preoperative nivolumab combination chemotherapy was administered for only one cycle owing to adverse events; however, a significant treatment effect was achieved. Therefore, this case report suggests that even if the patient is unable to continue preoperative treatment, it is important not to miss the possibility of surgery, as a favorable treatment effect may have been achieved.

## Supplementary Information

Additional file 1. Supplementary Fig. 1 Intraoperative findings of interlobar pulmonary artery and branches of A^1+2c^ and A^4+5^ (**A**), superior pulmonary vein (**B**), and main pulmonary artery and branch of A^3^ + A^1+2a+b^ (C).

## Data Availability

Data sharing is not applicable to this article as no datasets were generated or analyzed during the current study.

## Abbreviations

CT: Computed tomography
ICI: Immune checkpoint inhibitor
NSCLC: Non-small cell lung cancer
EFS: Event-free survival
pCR: Pathological complete response
PS: Performance status
LUL: Left upper lobe
PET: Positron emission tomography
SUV-max: Standard uptake value-max
MPR: Major pathological response

## References

[1] Forde PM, Spicer J, Lu S, et al. Neoadjuvant nivolumab plus chemotherapy in resectable lung cancer. N Engl J Med. 2022;386:1973–85. 35403841 10.1056/NEJMoa2202170 PMC9844511

[2] Cornelissen R, Havel L, Karaseva N, et al. Perioperative nivolumab in resectable lung cancer. Epub ahead of print 2024. 10.1056/NEJMoa2311926. 10.1056/NEJMoa2311926 38749033

[3] Wakelee H, Liberman M, Kato T, et al. Perioperative pembrolizumab for early-stage non–small-cell lung cancer. N Engl J Med. 2023;389:491–503. 10.1056/NEJMoa2302983 PMC11074923 37272513

[4] Heymach JV, Harpole D, Mitsudomi T, et al. Perioperative durvalumab for resectable non–small-cell lung cancer. N Engl J Med. 2023;389:1672–84. 37870974 10.1056/NEJMoa2304875

[5] Wu Y, Verma V, Gay CM, et al. Neoadjuvant immunotherapy for advanced, resectable non-small cell lung cancer: a systematic review and meta-analysis. Cancer. 2023;129:1969–85. 36994945 10.1002/cncr.34755

[6] Banna GL, Hassan MA, Signori A, et al. Neoadjuvant chemo-immunotherapy for early-stage non-small cell lung cancer: a systematic review and meta-analysis. JAMA Netw Open. 2024;7:e246837. 10.1001/jamanetworkopen.2024.6837 PMC11022115 38625698

[7] Mountzios G, Remon J, Hendriks LEL, et al. Immune-checkpoint inhibition for resectable non-small-cell lung cancer - opportunities and challenges. Nat Rev Clin Oncol. 2023;20:664–77. 37488229 10.1038/s41571-023-00794-7

[8] Conroy MR, Dennehy C, Forde PM. Neoadjuvant immune checkpoint inhibitor therapy in resectable non-small cell lung cancer. Lung Cancer. 2023;183: 107314. 37541935 10.1016/j.lungcan.2023.107314

[9] Desai AP, Adashek JJ, Reuss JE, et al. Perioperative immune checkpoint inhibition in early-stage non-small cell lung cancer: a review. JAMA Oncol. 2023;9:135–42. 36394834 10.1001/jamaoncol.2022.5389

[10] Deutsch JS, Cimino-Mathews A, Thompson E, et al. Association between pathologic response and survival after neoadjuvant therapy in lung cancer. Nat Med. 2024;30:218–28. 37903504 10.1038/s41591-023-02660-6 PMC10803255

[11] Forde PM, Chaft JE, Smith KN, et al. Neoadjuvant PD-1 blockade in resectable lung cancer. N Engl J Med. 2018;378:1976–86. 29658848 10.1056/NEJMoa1716078 PMC6223617

[12] Aokage K, Shimada Y, Yoh K, et al. Pembrolizumab and ramucirumab neoadjuvant therapy for PD-L1-positive stage IB-IIIA lung cancer (EAST ENERGY). J Clin Oncol. 2023;41:8509.

[13] Kimura H, Araya T, Yoneda T, et al. Long-lasting responses after discontinuation of nivolumab treatment for reasons other than tumor progression in patients with previously treated, advanced non-small cell lung cancer. Cancer Commun. 2019;39:1–5. 10.1186/s40880-019-0423-3 PMC6873691 31753015

[14] Tachihara M, Negoro S, Inoue T, et al. Efficacy of anti-PD-1/PD-L1 antibodies after discontinuation due to adverse events in non-small cell lung cancer patients (HANSHIN 0316). BMC Cancer. 2018;18:1–6. 30285770 10.1186/s12885-018-4819-2 PMC6171229

