# Supplementary figures and images for: A stage IIIA lung adenocarcinoma case achieving pathological response with only one cycle of preoperative nivolumab combination chemotherapy
Source: Gen Thorac Cardiovasc Surg Cases. 2025 Feb 3;4:6. doi: 10.1186/s44215-025-00187-5 (PMC11792341; doi:10.1186/s44215-025-00187-5)

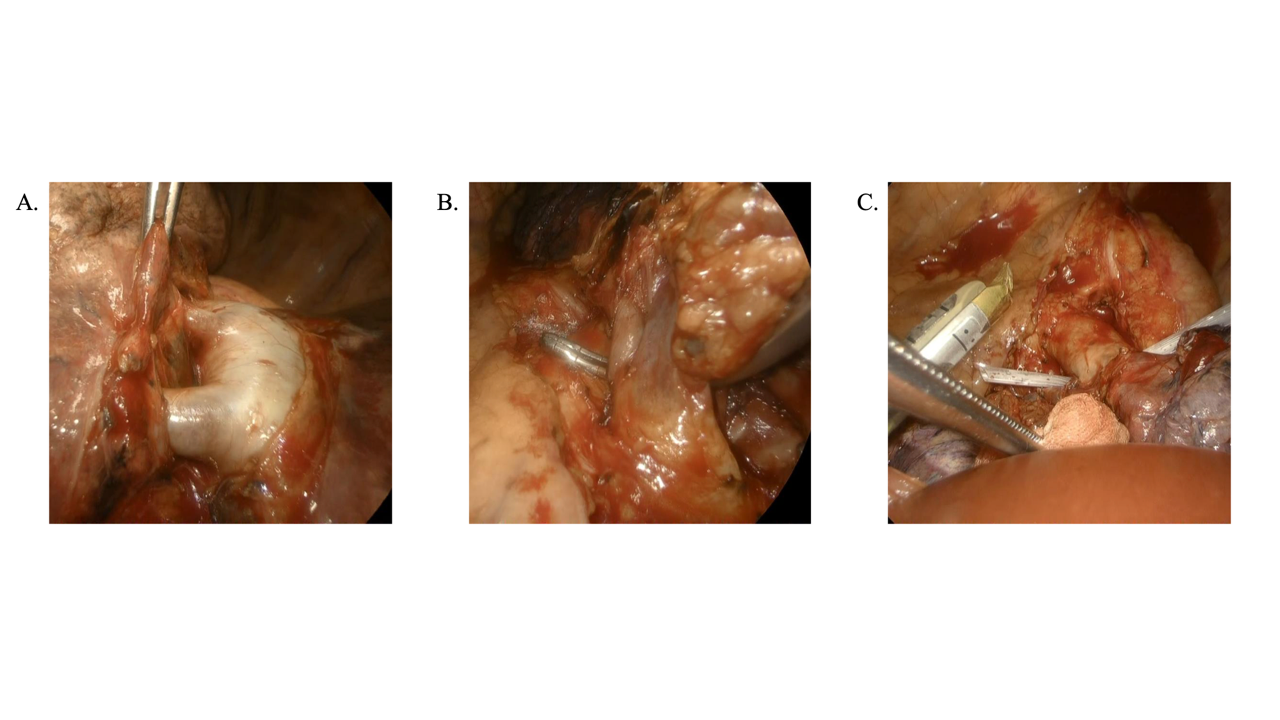

Supplement: Supplementary file 1 — Additional file 1. Supplementary Fig. 1 Intraoperative findings of interlobar pulmonary artery and branches of A1+2c and A4+5 (A), superior pulmonary vein (B), and main pulmonary artery and branch of A3 + A1+2a+b (C). [file 44215_2025_187_MOESM1_ESM.tiff]
